# Exploring Step‐by‐Step Assembly of Nanoparticle:Cytochrome Biohybrid Photoanodes

**DOI:** 10.1002/celc.201700030

**Published:** 2017-05-15

**Authors:** Ee Taek Hwang, Katherine L. Orchard, Daisuke Hojo, Joseph Beton, Colin W. J. Lockwood, Tadafumi Adschiri, Julea N. Butt, Erwin Reisner, Lars J. C. Jeuken

**Affiliations:** ^1^ School of Biomedical Sciences, and The Astbury Centre for Structural Molecular Biology University of Leeds Leeds LS2 9JT U.K; ^2^ Department of Chemistry University of Cambridge Lensfield Road Cambridge CB2 1EW U.K.; ^3^ Advanced Institute for Materials Research Tohoku University 2-1-1 Katahira Aoba-ku Sendai Miyagi 980-8577 Japan; ^4^ Centre for Molecular and Structural Biochemistry School of Chemistry, and School of Biological Sciences University of East Anglia Norwich Research Park Norwich NR4 7TJ United Kingdom

**Keywords:** artificial photosynthesis, biophotoelectrochemistry, bioelectrochemistry, dye-sensitized TiO_2_ nanoparticles, quantum dots

## Abstract

Coupling light‐harvesting semiconducting nanoparticles (NPs) with redox enzymes has been shown to create artificial photosynthetic systems that hold promise for the synthesis of solar fuels. High quantum yields require efficient electron transfer from the nanoparticle to the redox protein, a property that can be difficult to control. Here, we have compared binding and electron transfer between dye‐sensitized TiO_2_ nanocrystals or CdS quantum dots and two decaheme cytochromes on photoanodes. The effect of NP surface chemistry was assessed by preparing NPs capped with amine or carboxylic acid functionalities. For the TiO_2_ nanocrystals, binding to the cytochromes was optimal when capped with a carboxylic acid ligand, whereas for the CdS QDs, better adhesion was observed for amine capped ligand shells. When using TiO_2_ nanocrystals, dye‐sensitized with a phosphonated bipyridine Ru(II) dye, photocurrents are observed that are dependent on the redox state of the decaheme, confirming that electrons are transferred from the TiO_2_ nanocrystals to the surface via the decaheme conduit. In contrast, when CdS NPs are used, photocurrents are not dependent on the redox state of the decaheme, consistent with a model in which electron transfer from CdS to the photoanode bypasses the decaheme protein. These results illustrate that although the organic shell of NPs nanoparticles crucially affects coupling with proteinaceous material, the coupling can be difficult to predict or engineer.

## 1 Introduction

The utilization of solar energy for fuel production is one of the most promising sustainable and environmentally friendly processes.^1^, ^2^ In biology, photosynthesis converts light energy into chemical energy using numerous proteins and enzymes.^3^, ^4^, ^5^ The unique features of natural photosynthesis, especially its high quantum yield, are a source of inspiration for artificial solar fuel systems.^6^ Various biophotoelectrochemical strategies have been reported, which share their aim to exploit the high quantum yield of light harvesting proteins, such as photosystems I and II (PSI and PSII), but differ in their application, which ranges from biophotovoltaics,^7^, ^8^, ^9^, ^10^, ^11^, ^12^ optobioelectronics^13^ to fuel production^14^, ^15^, ^16^, ^17^ and water splitting.^18^, ^19^, ^20^ For the production of solar fuels, both the use of platinum,^16^, ^17^ organometalic catalysts^15^, ^18^ and redox enzymes^19^, ^20^ have been explored.

Despite significant progress over the last decade, purification of light‐harvesting proteins is laborious and expensive, while these proteins, especially PSII, have a limited lifetime under illumination conditions.^8^, ^20^ In an attempt to tackle this drawback, systems have been developed in which light is harvested by dyes, quantum dots (QDs) or dye‐sensitized semiconducting nanoparticles (DS‐SC‐NPs) and combined with redox biocatalysts to produce chemical fuels such as hydrogen or carbon‐based fuels.^21^, ^22^, ^23^ However, the latter systems suffer from inefficient electron transport and coupling of catalytic steps, leading to non‐productive charge separation and low quantum yields.

**Figure 1:**
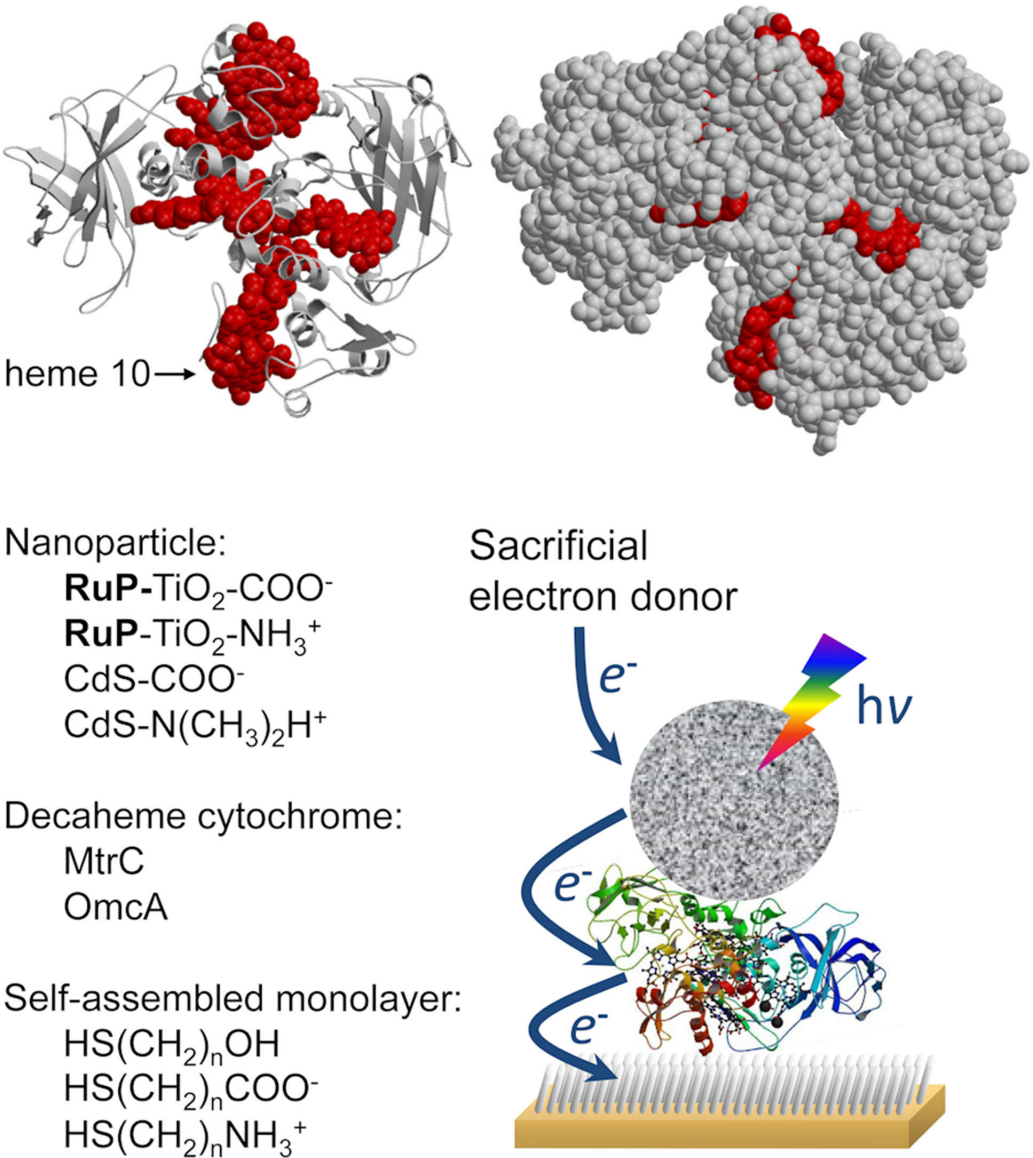
Top: The structure of MtrC (pdb code: 4lm8). The ten hemes are coloured in red. The space‐filling representation on the top right indicates that several hemes protrude through the protein's surface. The edge‐to‐edge distances between neighbouring hemes are less than 7 Å. Bottom: Schematic representation of the biohybrid photoanode system constructed on a gold electrode modified with a self‐assembled monolayer (SAM; not to scale). On the left, a list is given of the different semiconducting nanoparticles, decaheme cytochromes and SAM‐modified gold electrodes that were compared in this study.

Redox proteins such as cytochrome *c* and plastocyanin (a small copper‐containing protein) have been tested in an effort to improve the lifetime of the charge separated state and the overall quantum efficiency of these systems.^24^, ^25^, ^26^ For example, oxidized and reduced cytochrome *c* have been added in solution to photoelectrodes made up of monolayers of CdS QDs, enabling control over the photocurrent direction (cathodic or anodic) and amplifying photocurrents.^27^, ^28^ However, both cytochrome *c* and plastocyanin are relatively small proteins and contain only one redox active centre. As such, their ability to create a charge separated state is naturally limited. Furthermore, as only one redox active centre is present in these proteins, there are many ways in which QDs or DS‐SC‐NPs can bind to cytochrome *c* and plastocyanin without having an efficient electron transfer path to the redox‐active site. Finally, even if a charge separated state is generated, the electron will need a different pathway to transfer to an electrocatalyst for the synthesis of a chemical fuel. In other words, to improve the quantum yield, a redox protein needs to be employed that enables different entry and exit sites for electrons. The ideal properties of a redox protein in a photosystem would thus be that (a) the redox groups are accessible from different ‘faces’ or sides of the protein to allow charge transfer from NP to the electrocatalyst/electrode and (b) that multiple well‐connected redox groups are present such that a charge (i. e. the electron) can be rapidly separated from the QDs or DS‐SC‐NPs. In these respects, the family of decaheme proteins from species of *Shewanella* 29, 30 present themselves as attractive candidates, with well‐aligned redox sites that would be very hard to emulate in synthetic compounds. Their hemes are arranged as a staggered cross in which eight of the hemes form an approx. 7 nm wire that spans the protein and contacts the surface at both termini, Figure 1 top. A separate tetraheme wire extends in an orthogonal direction, also contacting the protein surface at both termini. The neighbouring hemes are positioned in close proximity with reduction potentials and electronic couplings that are optimal for the very rapid intraprotein electron exchange that has been observed experimentally.^31^, ^32^ These decaheme proteins are located on the extracellular side of the outer membrane, and basic studies into the decaheme proteins might enable in vivo photocatalytic systems in the future.

In order to test if these decaheme cytochromes have the ability to efficiently ‘extract’ electrons from light‐excited QD and create a beneficial charge separated state, we have previously reported^33^ on the assembly of a bio‐hybrid system composed of a dye‐sensitized TiO_2_ nanocrystal (**RuP**‐TiO_2_) coupled to an underlying anode via the decaheme protein MtrC from *Shewanella oneidensis* MR‐1. These photoanodes were prepared via a simple two‐step process: protein adsorption on a gold electrode that was modified with a self‐assembled monolayer (SAM), followed by immobilization of particles onto the MtrC film, forming double layer structures as schematically shown in Figure 1. In this system, MtrC acted as an electron‐transfer conduit between negatively charged **RuP**‐TiO_2_ nanocrystals and the underlying anode.^33^

Here, we have extended this approach by comparing how the surface of the NP and the identity of the cytochrome affect chemical interactions, electronic coupling and stability of the hybrid system. Alongside MtrC, a second decaheme cytochrome, OmcA, was investigated. OmcA displays structural homology to MtrC and both are outer membrane associated c‐type cytochromes, which play an important role in the ability of *S. oneidensis* to transfer electrons extracellularly to minerals, as part of their anaerobic respiration.^34^ OmcA is less charged than MtrC (estimated pIs are 6.2 and 5.6, respectively) and hence OmcA was included in this study to test the effect of protein charge on the interaction with QDs and DS‐SC‐NPs. NPs with TiO_2_ or CdS cores and ligand shells containing either amine or carboxylic acid ‘head groups’ were compared to investigate the impact of NP chemistry and charge in the hybrid system. The ligand shells altered the interactions between the NPs and the decaheme cytochromes and, consequently, affected photo‐induced electron transfer behaviour. This study provides insights into the interaction between nanomaterials and redox proteins, a fundamental feature of future biohybrid electrochemical cell designs.

## 2 Results and Discussion

### 2.1 Synthesis and Characterization of RuP‐TiO_2_ and CdS NPs

We previously reported on the synthesis and characterisation of oleic acid‐modified TiO_2_ nanocrystals and the replacement of the oleic acid shell with 3,4‐dihydroxybenzoic acid (DHBA) in a two‐step procedure to create TiO_2_‐DHBA (hereafter named TiO_2_‐COO^−^).^33^ To functionalize the TiO_2_ nanocrystals with amine‐capped ligands (TiO_2_‐NH_3_^+^), the oleic acid shell was instead replaced with 3‐hydroxyacetaminophen (3HAP), which is subsequently hydrolysed to 3,4‐dihydroxyaniline (DHA). FT‐IR spectra of the TiO_2_‐NH_3_^+^ displayed N−H bending vibration at ∼1600 cm^−1^ confirming ligand exchange from oleic acid to 3HAP and subsequent hydrolysis to DHA (Figure S1). No change in crystallinity or particle size occurred during ligand exchange.

The size of the TiO_2_‐NH_3_^+^ nanocrystals after synthesis and the ligand‐exchange process was determined to be 5.8±1.0 nm by TEM (Figure S2), similar to the previously determined size of TiO_2_‐COO^−^ (6.8±0.7 nm). The amount of DHA on the TiO_2_‐NH_3_^+^ nanocrystals was quantified by thermogravimetric analysis (Figure S3) and found to be 12.27 % (w/w) compared to 7.26 % previously determined for TiO_2_‐COO^−^.^33^ Considering the size of the TiO_2_‐NH_3_^+^ nanocrystals (calculated surface area 107±37 nm^2^ and volume 105±53 nm^3^) and the molecular weight of DHA (125.13 g mol^−1^) and density of anatase (3.8 g cm^−3^), the number of DHA molecules attached to the nanocrystals is estimated to be 2.5 nm^−2^ (1.4 nm^−2^ for TiO_2_‐COO^−[33]^). Zeta‐potential measurements (Figure S4) showed a pI at pH 5.1, which is lower than initially expected for an amine modified NPs. However, the p*K*_a_ of any aniline is very sensitive to its ring substituents and chemical surrounding, where the p*K*_a_ of a conjugated acid of aniline is 4.9, much lower than for aminophenol. Furthermore, the surface of the TiO_2_ core could significantly contribute to the zeta‐potential (the zeta‐potential of TiO_2_ NPs has previously been shown to strongly depend on its shape and synthesis^35^). Combined with the FT‐IR analysis, we conclude that the 3HAP ligand is hydrolyzed to DHA on the TiO_2_ nanocrystals. The zeta‐potential of TiO_2_‐COO^−^ was previously determined to be 4.5.^33^ At pH 7, the zeta potential of the TiO_2_‐COO^−^ and TiO_2_‐NH_3_^+^ are similar at −34±5 and −20±5 mV, respectively.

Dye‐sensitization of the TiO_2_‐NH_3_^+^ nanocrystals was carried out as previously described for TiO_2_‐COO^−^, which was modified with a phosphonated bipyridine Ru(II) dye (**RuP**).^33^ The quantity of **RuP** adsorbed to the TiO_2_‐NH_3_^+^ particles (herein referred to as **RuP**‐TiO_2_‐NH_3_^+^) was estimated by UV‐vis spectroscopy to be 48±19 nmol (mg TiO_2_)^−1^, which is less than for **RuP**‐TiO_2_‐COO^−^ (90±20 nmol mg^−1^). In spite of this difference, the adsorption of **RuP** has a similar effect on the zeta‐potential for both particles, which are raised by about 10 to 15 mV (to −23±5 and −12±5 mV for **RuP**‐TiO_2_‐COO^−^ and RuP‐TiO_2_‐NH_3_^+^, respectively, at pH 7). The **RuP**‐TiO_2_‐NH_3_^+^ displayed characteristic phosphonate resonances from **RuP** in the FT‐IR spectrum of a dried sample (Figure S1).

Oleic acid‐capped CdS QDs (CdS‐OA) were prepared by a hot injection method and used to prepare water soluble QDs with positive or negative surface charge by ligand exchange with either 2‐(dimethylamino)ethanethiol (CdS‐N(CH_3_)_2_H^+^) or 3‐mercaptopropionic acid (CdS‐COO^−^). Ligand exchange was confirmed by FT‐IR (Figure S5), and no change in either the crystal phase (XRD, Figure S6) or particle size (*d*=4.4 nm; based on UV‐vis absorption *λ*_max_=430 nm; Figure S7) was observed.^36^ The zeta potentials of the particles at pH 7 was found to be +38±2 and −39±6 mV for CdS‐N(CH_3_)_2_H^+^ and CdS‐COO^−^, respectively, reflecting the different ligand shells of the particles.

### 2.2 Characterization of Decaheme Protein Films

**Figure 2:**
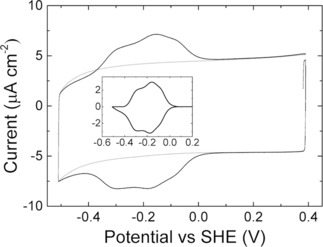
CV at 1 V s^−1^ and 20 °C before (grey) and after (black) adsorption of OmcA on a gold electrode modified with a SAM of 6‐OH/6‐NH_3_^+^ (ratio of 80/20) in aqueous buffer solution (20 mM MOPS, 30 mM Na_2_SO_4_ at pH 7.4). The insert shows the baseline‐subtracted redox peak maxima.

The redox properties of the cytochrome films were explored by cyclic voltammetry (CV). No redox signals were observed for MtrC or OmcA adsorbed on negatively‐charged gold electrodes (i. e., modified with SAMs baring carboxylic acid headgroups, either pure or mixed with alcohol terminated alkanethiols). In contrast, on either positively charged or neutral SAMs, oxidation and reduction signals between −400 to 0 mV were observed for OmcA (Figure 2), similar to that published previously for MtrC.^37^, ^38^ This is expected as, at neutral pH, OmcA, like MtrC, exhibits an overall negative charge (Figure S8) although OmcA has a higher estimated isoelectric point (estimated pIs are 6.2 and 5.6 for OmcA and MtrC, respectively). Furthermore, the positively charged SAM is expected to interact favourably with the negatively charged propionate groups of the c‐type hemes, leading to the desired orientation where electrons are rapidly exchanged with the electrode. Indeed, the cyclic voltammograms display almost fully reversible redox signals even at 1000 mV s^−1^ scan rate, indicating fast interfacial electron transfer rates with *k*_0_ values >100 s^−1^ (*k*_0_ is the electron transfer rate at zero over‐potential). We further note that the shape of the redox signals do not significantly change upon changing the scan rate, while the normalised peak area (i. e., electroactive coverage of the heme groups, see below) remains unaltered up to scan rates of 1000 mV s^−1^. This is consistent with the expectation that the electron exchange between the hemes is also very fast, i. e. >100 s^−1^ as reported previously^32^ and predicted by computation.^31^

The highest electroactive coverage for both OmcA and MtrC was achieved using SAMs consisting of an 80/20 mixture of alcohol and amine terminated alkanethiols, although the optimal length of the alkyl chain differed slightly for the two decaheme cytochromes (Table 1). It is unclear why the chain length of alkane thiols in the SAM affects OmcA and MtrC differently. We hypothesize that shorter chain lengths might result in a more fluidic or disordered SAM,^39^, ^40^ which, for OmcA, appears to be beneficial.

**Table 1:** Coverage of OmcA and MtrC on different SAM‐modified gold electrodes as determined by QCM−D and CV.

	OmcA film			MtrC film		
SAM^[c]^	Coverage (QCM−D) [pmol cm^−2^]	Electroactive coverage (CV) [pmol cm^−2^]	Ratio^[a]^	Coverage (QCM−D) [pmol cm^−2^]	Electroactive coverage (CV) [pmol cm^−2^]	Ratio^[a]^
20 % 8‐NH_3_^+^/80 % 8‐OH	3.7±0.3	0.3±0.1	0.07	3.6±0.2^33^	0.17±0.02^33^	0.05^33^
20 % 6‐NH_3_^+^/80 % 6‐OH	3.7±0.2	0.9±0.1	0.24	3.6±0.2	^[b]^	^[b]^

[a] Ratio is taken from the coverage as observed with CV (electroactive coverage) and QCM−D (CV/QCM−D). [b] The electroactive coverage was highly variable between different electrodes, which prevented its accurate determination. [c] 8‐NH_3_^+^=8‐mercaptooctylamine; 6‐NH_3_^+^=6‐mercaptohexylamine; 8‐OH=8‐mercaptooctanol; 6‐OH=6‐mercaptohexanol.

Based on the peak area, the electroactive coverage (Γ_ea_) can be quantified according to Γ_ea_=(peak area)/*nFAυ*, where *n* is the number of electrons (10 for OmcA and MtrC), *F* the Faraday constant, *A* the electrode area (0.25 cm^2^) and υ is the scan rate. It is immediately obvious from Table 1 that Γ_ea_ is much higher for OmcA compared to MtrC.

We note that the engineered OmcA and MtrC constructs were generated from plasmids that code for a C‐terminal enterokinase protease sequence (DDDDK) that could provide an additional negative charge close to the surface exposed heme 10 (see Figure 1 and S8 for the position of heme 10). LC–MS indicated that MtrC has undergone C‐terminal degradation and in some cases the preparations showed heterogeneity. In cases where heterogeneity was observed, the mass difference between major and minor species correlated with the mass of the negatively charged aspartic acid sequence (Figure S9). Direct electrochemical comparison of different MtrC preparations showed that heterogeneous samples (containing protein of lower molecular weight) were ‘electro‐silent’ and did not display any significant redox signals with cyclic voltammetry (Γ_ea_<0.02 pmol cm^−2^). This correlation between the LC–MS and CV results suggests that the aspartic acid residues at the C‐termini of the engineered MtrC (and the engineered OmcA, which has an identical engineered C‐terminus) play a crucial role in orienting the decaheme on the gold electrode.

**Figure 3:**
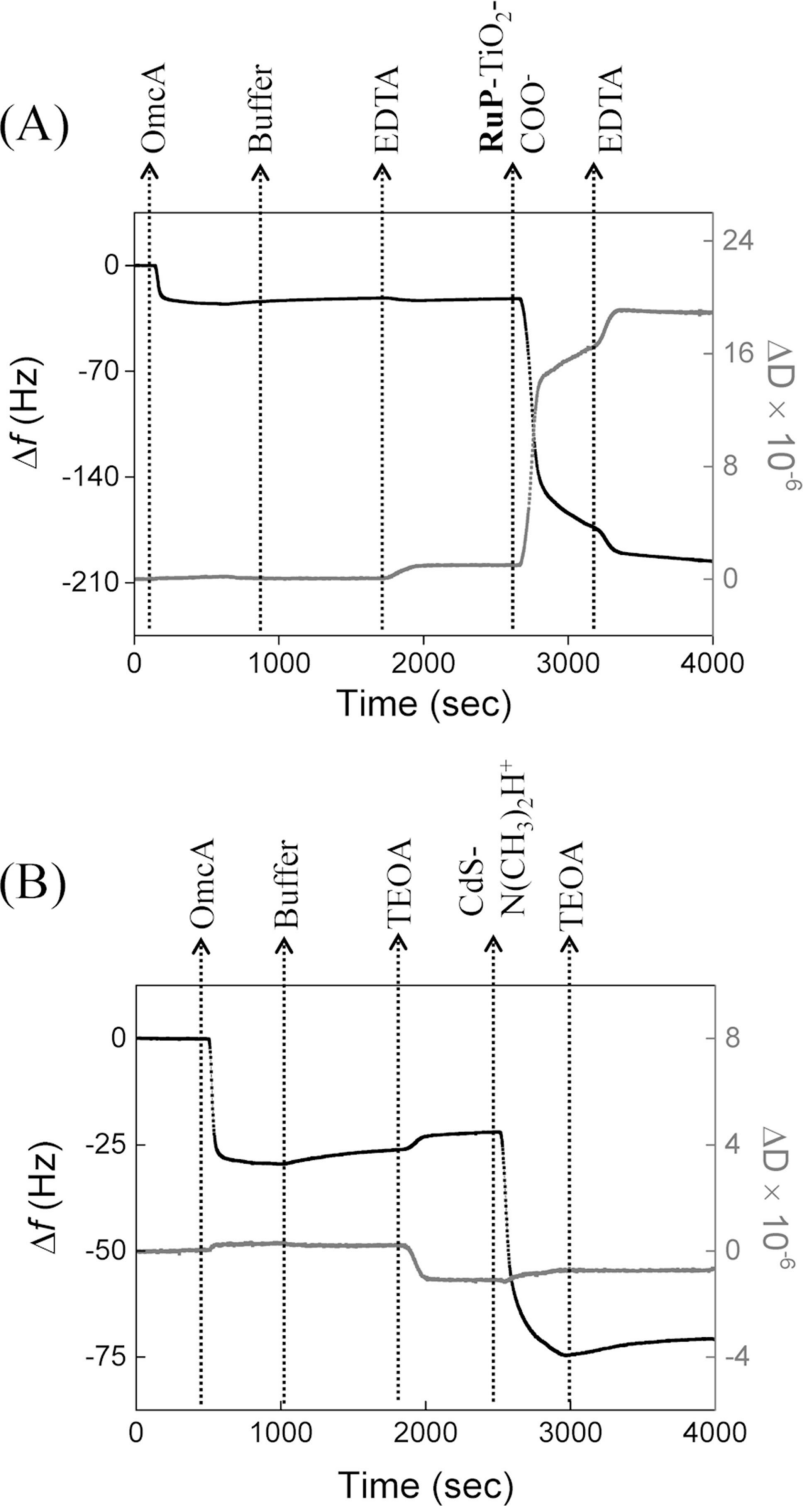
QCM−D results with frequency (black line, left axis) and dissipation (grey line, right axis) against time for a gold crystal at 21 °C. The gold surface has been modified with 6‐OH/6‐NH_3_^+^ (ratio 80/20) SAM prior to the experiments. The plots shown are representative of triplicate experiments. As indicated, the gold‐coated QCM−D crystal is consecutively incubated with: A) OmcA (1 μM) in buffer (20 mM MOPS, 30 mM Na_2_SO_4_ at pH 7.4); buffer only; EDTA (25 mM EDTA at pH 7.4); **RuP**‐TiO_2_‐COO^−^ (0.2 mg mL^−1^) in EDTA and, finally, EDTA. B) OmcA (1 μM) in buffer (20 mM MOPS, 30 mM Na_2_SO_4_ at pH 7.4); buffer only; TEOA (25 mM TEOA at pH 7.4); CdS‐N(CH_3_)_2_H^+^ (0.2 mg mL^−1^) in TEOA and, finally, TEOA.

The coverage of decaheme cytochromes on the modified gold was also characterized using a quartz‐crystal microbalance with dissipation (QCM−D, Figure 3A), which provides information on both the mass and viscoelastic properties of the adsorbed protein layer. Small dissipation values were observed upon immobilization of OmcA/MtrC, indicating that these decaheme proteins form rigid films on the surface and enabling the use of the Sauerbrey equation to estimate the coverage. If it is assumed that the mass of proteins is increased by 25 % due to tightly bound water,^41^ the coverages by QCM−D are estimated to be 3.6–3.7 pmol cm^−2^ for both proteins and the different SAM surfaces studied (Table 1). OmcA and MtrC have similar molecular weight (83 and 75 kDa, respectively) and dimensions (9.5x6.0x5.0 nm^3^).^29^, ^30^ Depending on the orientation of the decaheme proteins, a closely packed monolayer would consist of 5.5 pmol cm^−2^ (upright orientation) or 2.9 pmol cm^−2^ (prone orientation) and thus the QCM−D data indicate well packed monolayers. The similarity in QCM−D response is in stark contrast to the electroactive coverage, which is very different for the two proteins (Table 1).

Further characterization as described below was performed with 6‐mercapto‐hexanol (6‐OH)/6‐mercapto‐hexylamine (6‐NH_3_^+^) at 80/20 ratio SAMs for OmcA films and 8‐mercapto‐octanol (8‐OH)/8‐mercapto‐octylamine (8‐NH_3_^+^) at 80/20 ratio for MtrC films, as these systems give rise to the highest electroactive coverages.

### 2.3 Characterization of Decaheme Cytochrome/RuP‐TiO_2_ and Decaheme Cytochrome/CdS Films

QCM−D was also used to quantify the adsorption of **RuP**‐TiO_2_ and CdS NPs directly on either SAMs or on the OmcA/MtrC protein films (Figure 3). Addition of **RuP**‐TiO_2_‐COO^−^ results in a rapid decrease in frequency and a rise in the dissipation. Based on the Sauerbrey equation, for **RuP**‐TiO_2_‐COO^−^ nanocrystals (6.8±0.7 nm diameter), we estimate coverages of 7.0±0.2 pmol cm^−2^ and 6.2±0.2 pmol cm^−2^ on the OmcA film and SAM surface, respectively (Table 2; NP coverages are given in number of particles). A well‐packed monolayer of TiO_2_ nanocrystals (assuming a hexagonal packing of perfect spheres of 6.8 nm diameter) equates to 4.2 pmol cm^−2^, indicating that there might be some aggregation of the **RuP**‐TiO_2_‐COO^−^ on the OmcA surface. Following **RuP‐**TiO_2_‐COO^−^ adsorption, the electroactive coverage of both MtrC and OmcA, as determined by CV, are decreased by ∼70 %, which we attribute to reorientations within the protein films, altering the electronic coupling with the electrode. We note that a reduction in electroactive coverage could also be explained by either desorption or denaturation of OmcA/MtrC. However, the photoelectrochemical response of the **RuP**‐TiO_2_‐COO^−^ systems are more consistent with our hypothesis of a reorientation of the protein film (see discussion below).

**Table 2:** Total and electroactive coverage of different types of NPs/decaheme cytochrome double layers on gold electrodes modified with different SAMs. Coverage determined by QCM−D and electroactive coverages determined by cyclic voltammetry.

Decaheme	Nanoparticles	Coverage of NPs (QCM−D)^[c]^ [pmol cm^−2^]	Electroactive coverage of OmcA/MtrC without NPs (CV) [pmol cm^−2^]	Electroactive coverage of OmcA/MtrC with NPs (CV) [pmol cm^−2^]	Ratio^[a]^
	**RuP**‐TiO_2_‐COO^−^	7.0±0.2	0.9±0.1	0.3±0.1	0.3
OmcA film	**RuP**‐TiO_2_‐NH_3_^+^	1.0±0.2	0.9±0.1	n.d.	–
(20 % 6‐NH_3_^+^/80 % 6‐OH)	CdS‐N(CH_3_)_2_H^+^	4.7±0.2	0.9±0.1	0	0
	CdS‐COO^−^	0.1±0.01	0.9±0.1	0	0
	**RuP**‐TiO_2_‐COO^−^	3.3±0.1^[b]^	0.17±0.02^[b]^	0.07±0.02^[b]^	0.4^[b]^
MtrC film	**RuP**‐TiO_2_‐NH_3_^+^	0.5±0.1	0.17±0.02^[b]^	n.d.	–
(20 % 8‐NH_3_^+^/80 % 8‐OH)	CdS‐N(CH_3_)_2_H^+^	4.3±0.2	0.17±0.02^[b]^	0	0
	CdS‐COO^−^	0.2±0.01	0.17±0.02^[b]^	0	0

[a] Ratio is taken from the electroactive coverage before and after nanoparticle immobilization. [b] Data taken from reference^33^. [c] To calculate the coverage of NPs, it is assumed that upon adsorption of NPs on the decaheme film, no decaheme dissociates from the surface. n.d.=no data.

Next, we tested whether the surface chemistry of the **RuP**‐TiO_2_ nanocrystals influences their interactions with OmcA/MtrC. As OmcA and MtrC are negatively charged without any significant surface regions that are positive (Figure S8), we expected **RuP**‐TiO_2_‐NH_3_^+^ to show improved adherence. Unexpectedly, however, the coverage of the **RuP**‐TiO_2_‐NH_3_^+^ nanocrystals is much lower on both OmcA or MtrC (Table 2). Less than 25 % of a dense monolayer coverage was obtained (compared to 166 % for TiO_2_‐COO^−^), while, upon illumination, much lower photocurrents were observed (see below and Table 3).

**Table 3:** Photoelectrochemical properties of different types of NPs photoanodes and NPs/decaheme photoanodes.^[a]^

		Photocurrent [nA cm^−2^]		Photocurrent/NPs [electrons s^−1^]
Decaheme	Nanoparticles	Without decaheme	With decaheme	With decaheme
	**RuP**‐TiO_2_‐COO^−^	500	450	≈0.8
OmcA film	**RuP**‐TiO_2_‐NH_3_^+^	150	100	≈1.5
(20 % 6‐NH_3_^+^/80 % 6‐OH)	CdS‐N(CH_3_)_2_H^+^	600	480	≈1.5
	CdS‐COO^−^	160	150	≈15
	**RuP**‐TiO_2_‐COO^−^	500^[b]^	500^[b]^	≈1.5
MtrC film	**RuP**‐TiO_2_‐NH_3_^+^	50	50	≈1
(20 % 8‐NH_3_^+^/80 % 8‐OH)	CdS‐N(CH_3_)_2_H^+^	670	500	≈1.5
	CdS‐COO^−^	240	150	≈12

[a] Photocurrents were measured at 0.4 V vs. SHE with either 25 mM EDTA (**RuP**‐TiO_2_) or 25 mM TEOA (CdS) as sacrificial electron donor. [b] Data taken from reference [33].

To further characterize the influence of NPs on their interaction with OmcA/MtrC, a second NP was studied. CdS NPs were synthesized with either negatively charged surface ligand (CdS‐COO^−^) or positive‐charged surface ligands (CdS‐N(CH_3_)_2_H^+^). Using QCM−D, the surface coverage of CdS‐N(CH_3_)_2_H^+^ nanoparticles was estimated to be 4.7±0.2 pmol cm^−2^ and 6.3±0.3 pmol cm^−2^ on the OmcA film and SAM surface, respectively, translating to ∼50 % and ∼65 % of a monolayer coverage (Figure 3B). Similar coverages were observed for MtrC (Table 2). CdS‐COO^−^ shows only 5–10 % of a theoretical monolayer coverage on either the protein or SAM surfaces (Table 2). Importantly, after CdS‐N(CH_3_)_2_H^+^ immobilization, the electroactive OmcA/MtrC coverages almost completely disappeared, which indicates that most decaheme cytochromes molecules are either reoriented or replaced by the CdS. The QCM−D traces do not give any indication that the decaheme cytochromes dissociate upon incubation with CdS, suggesting that if the CdS replaces the proteins on the surface, the proteins still remain attached the CdS NP. Interestingly, the dissipation signal in the QCM−D experiments points towards a different interaction for CdS compared to **RuP**‐TiO_2_‐COO^−^. Higher energy dissipation is due to a high viscoelastic coupling to the surface, thus the higher dissipation signal obtained for the **RuP**‐TiO_2_‐COO^−^ particles suggests a much less rigid binding to the protein decaheme layer (Figure 3).

### 2.4 Photoelectrochemical Response of Decaheme/RuP‐TiO_2_‐COO^−^ and Decaheme/CdS‐N(CH_3_)_2_H^+^

**Figure 4:**
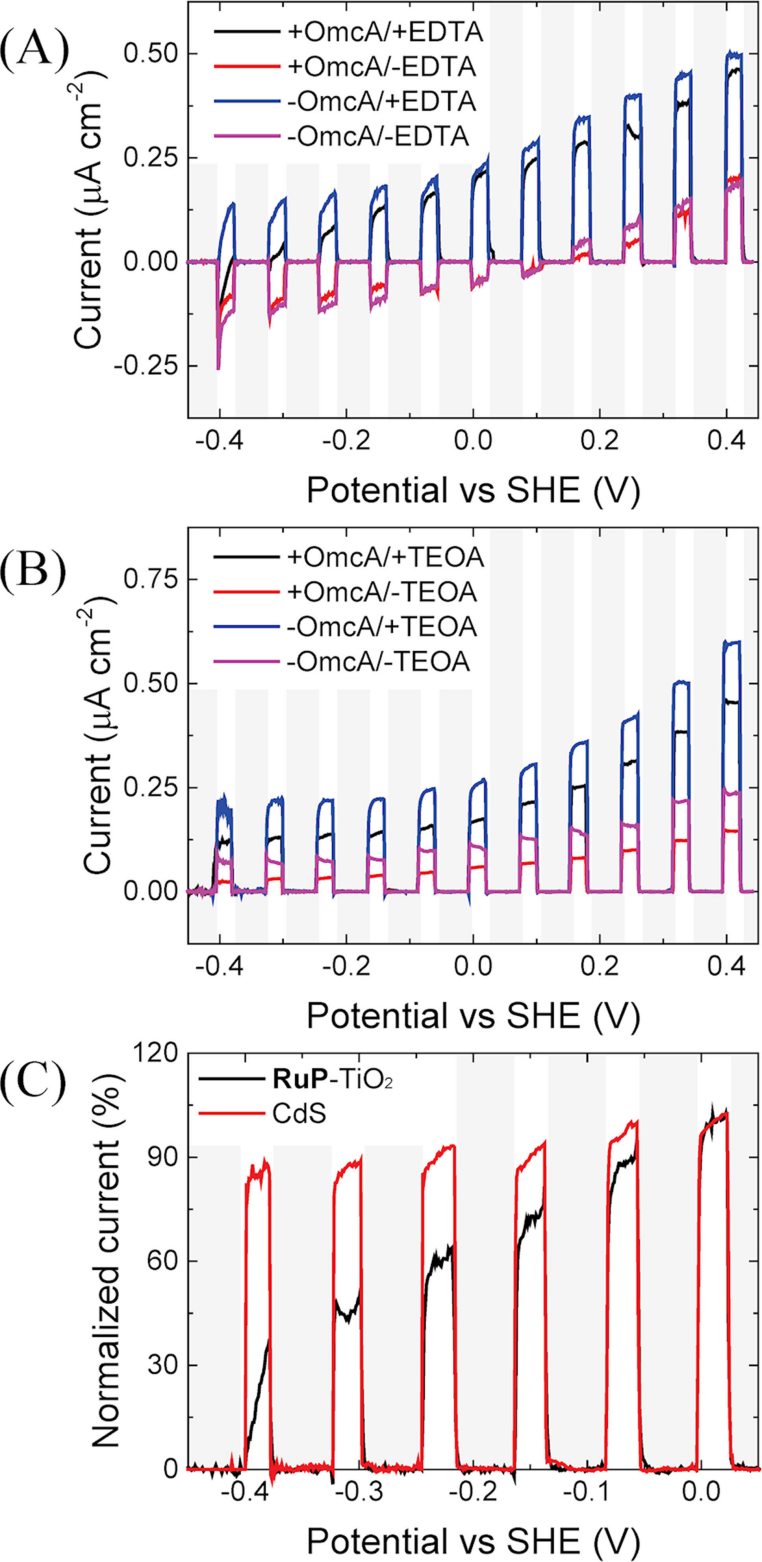
Effect of applied bias potential on the photocurrent of A) OmcA/**RuP**‐TiO_2_‐COO^−^ (+OmcA) and **RuP**‐TiO_2_‐COO^−^ only (−OmcA) measured with 25 mM EDTA (+ EDTA) and without EDTA (‐EDTA) and B) OmcA/CdS‐ N(CH_3_)_2_H^+^ (+OmcA) and CdS‐N(CH_3_)_2_H^+^ only (−OmcA) measured with 25 mM TEOA (+TEOA) and without TEOA (−TEOA), and C) normalized photocurrent (the difference between photocurrent generalized with and without the sacrificial electron donor) of OmcA/**RuP**‐TiO_2_‐COO^−^ and OmcA/CdS‐N(CH_3_)_2_H^+^, as indicated. The response was measured by linear sweep voltammetry (LSV) at 5 mV s^−1^. The LSVs are baseline subtracted, where the baseline is determined during the dark periods, which are indicated by grey shaded areas, and extrapolated to the illuminated periods.

**Figure 5:**
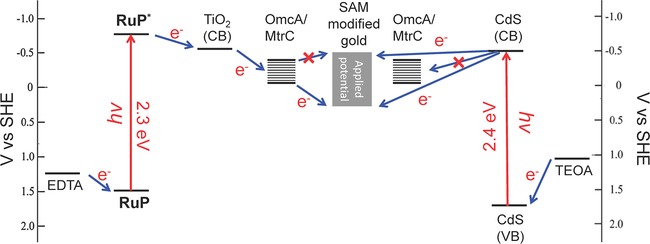
Energy diagrams of decaheme/**RuP**‐TiO_2_‐COO^−^ (left) and decaheme/CdS‐N(CH_3_)_2_H^+^ (right) photoelectrodes. The electron‐transfer reactions that have not been experimentally observed are indicated with a red cross through the blue arrows. The bottom and top line of the decaheme (OmcA/MtrC) indicate the span of reduction potentials of the hemes in the decaheme cytochromes. The bottom and top lines of the SAM modified gold electrodes indicates the potential window of the linear voltammetry scans used to measure the photocurrents (Figure 4). CB=conduction band. VB=valence band.

Upon illumination, in the presence of EDTA as a sacrificial electron donor, photocurrents are readily observed upon excitation of the **RuP**‐TiO_2_‐COO^−^ surfaces. This is shown here for the OmcA/**RuP**‐TiO_2_‐COO^−^ system (Figure 4) and previously reported for the MtrC/**RuP**‐TiO_2_‐COO^−^ system.^33^ For the OmcA/**RuP**‐TiO_2_‐COO^−^ system, the oxidative photocurrents at +400 mV are in the order of 450 nA cm^−2^ at a light intensity of 0.2 W cm^−2^ (Figure 4A). Importantly, the photocurrent diminishes to background level (i. e., to that without the sacrificial electron donor EDTA) when the potential is decreased from −200 to −400 mV (Figure 4A and C). In this potential range, OmcA and MtrC are reduced and unable to accept an electron from the photo excited **RuP**‐TiO_2_ (Figure 5). These results confirm that photo‐induced electron transfer proceeds through OmcA/MtrC. As noted above, upon adsorption of **RuP**‐TiO_2_‐COO^−^, the electroactive coverage of OmcA and MtrC was reduced by 70 % (Table 2), which we ascribe to a reorientation of some of the decaheme cytochrome molecules, reducing the interfacial electron transfer rate to such an extent that their electrochemical signal is no longer observed by CV.

We have previously reported that for the MtrC/ **RuP**‐TiO_2_‐COO^−^ system the photocurrent is linearly dependent on the light intensity up to 0.2 W cm^−2^.^33^ Furthermore, no strong effect was observed when changing the type of sacrificial electron donor or their concentration (besides EDTA, TEOA and ascorbic acid were compared with concentrations up to 100 mM). As also no difference is observed between MtrC and OmcA (Table 3), we propose that the photocurrent is limited by the absorption cross‐section of the **RuP**‐TiO_2_‐COO^−^ particles and that the interfacial electron transfer steps (from EDTA to **RuP** to TiO_2_ to decaheme cytochrome/anode) are not rate limiting. It can be calculated that about 0.8–1.5 electrons s^−1^ per **RuP**‐TiO_2_ particle are photogenerated, irrespective of the decaheme cytochrome conduit layer or whether the particles have a DHBA (‐COO^−^) or DHA (‐NH_3_^+^) ligand shell.

The magnitude of photocurrents for the CdS‐N(CH_3_)_2_H^+^ are very similar to those obtained with **RuP**‐TiO_2_‐COO^−^ nanocrystals (Figure 4B), although for the CdS NPs higher photocurrents were observed with TEOA than with EDTA as sacrificial electron donor and results with TEOA and CdS NPs are presented here. Importantly, however, no switching behaviour is observed with CdS‐N(CH_3_)_2_H^+^ when the potential is decreased to <−200 mV (Figure 4B and C), suggesting that electron transfer proceeds directly from CdS to the electrode, thereby bypassing the decaheme cytochrome conduit. This is consistent with the fact that the electroactive coverage of decaheme cytochrome disappears completely upon incubation with CdS‐N(CH_3_)_2_H^+^ (Table 2). As mentioned above, QCM−D data do not indicate that the decaheme cytochrome dissociates upon adsorption of the CdS NPs (Figure 4B) and we propose that CdS displaces the decaheme cytochromes on the surface, but that the decahemes remain bound to the CdS particles.

## 3 Conclusions

The OmcA/**RuP**‐TiO_2_‐COO^−^ system, like the MtrC/**RuP**‐TiO_2_‐COO^−^ system, exhibits a photo‐switching behaviour that confirms electron transfer via the OmcA/MtrC conduit. After illumination in the presence of a sacrificial electron donor, the OmcA/**RuP**‐TiO_2_‐COO^−^ and MtrC/**RuP**‐TiO_2_‐COO^−^ systems exhibit comparable photocurrents. The fact that the photocurrent is linearly dependent on the light intensity and that the type of sacrificial electron donor does not affect the magnitude of the photocurrent, suggest that photocurrent is limited by the ability of the thin layer of the **RuP**‐TiO_2_ particles to absorb light.

In contrast to the **RuP**‐TiO_2_ system, the photocurrent generated by CdS‐N(CH_3_)_2_H^+^ in the biohybrid system is not dependent on the redox state of either OmcA or MtrC, suggesting direct electron transfer from CdS to the electrode, bypassing the decaheme proteins. This difference in behaviour is assigned to CdS, which likely displaces OmcA/MtrC on the electrode surface. The stark differences between the CdS and TiO_2_ system clearly illustrate that despite advances in nanotechnology and methods to bioconjugate nanoparticles, it is still difficult to predict or engineer efficient electron transfer between nanoparticle photosensitizers and redox proteins. The successful construction of the biohybrid system (schematically shown in Figure 1) clearly requires a favourable interaction between the NP and the protein layer, but equally important is that the interaction between the NPs and the electrode surface is weak enough to not outcompete the electrostatic interaction between protein and electrode. The optimal interaction between **RuP**‐TiO_2_ and decaheme cytochromes is currently being developed as biological‐friendly photosensitizers for solar fuel production.

## **Experimental Section**

### Materials

4‐Morpholine propane sulfonic acid (MOPS), 4‐(2‐hydroxyethyl) piperazine‐1‐ethanesulfonic acid (HEPES), sodium sulfate, ethylenediaminetetraacetic acid (EDTA) disodium salt dehydrate, triethanolamine (TEOA), 8‐mercaptooctanol, 6‐mercaptohexanol, 8‐amino‐1‐octanethiol hydrochloride, ethanol, 6‐amino‐1‐hexanethiol hydrochloride, cadmium oxide (99.998 %), octadecene (ODE, 90 %), oleic acid (OA, 90 %), sulfur (99.998 %), 3‐mercaptopropionic acid (MPA, ≥99 %), 2‐(dimethylamino)ethanethiol hydrochloride (DMAET, 95 %), and tetramethylammonium hydroxide pentahydrate (TMAOH, 99 %) were purchased from Sigma Aldrich (UK); isopropanol, methanol, chloroform and dichloromethane were purchased from Fisher Chemicals; titanium tetraisopropoxide, oleic acid, hexadecylamine, and methyl 3,4‐dihydroxybenzoate were purchased from Wako Pure Chemical Industries Ltd; EPOTEK 307 was purchased from Epoxy technology. All reagents and solvents were used without any additional purification. [Ru(bpy)_2_(4,4’‐(PO_3_H_2_)_2_bpy)](Br)_2_ (**RuP**; bpy=2,2′‐bipyridine) was synthesized according to a literature procedure.^42^ The soluble forms of OmcA and MtrC were purified to >95 % purity as previously described.^29^, ^33^, ^43^ We note that the expression constructs of OmcA and MtrC contain an engineered C‐terminal poly‐histidine sequence, but the protein cannot be purified by conventional his‐tag affinity chromatography. LC–MS analysis (Figure S9) suggest that the engineered C‐terminal sequence is partially lost and terminates at or close to an included enterokinase protease sequence. Ultrapure water (Milli‐Q water, 18.2 MΩ cm) was used throughout.

### Synthesis of RuP‐TiO_2_ Nanocrystals

Dye‐sensitized TiO_2_ nanoparticles with either 3,4‐dihydroxyaniline (**RuP**‐TiO_2_‐NH_3_^+^) or 3,4‐dihydroxybenzoic acid (**RuP**‐TiO_2_‐COO^−^) functionalized surfaces were prepared from oleic‐acid capped TiO_2_ by first carrying out ligand exchange for the respective surface group, then mixing with the dye solution (**RuP**). The oleic acid‐modified TiO_2_ and **RuP‐**TiO_2_‐COO^−^ were synthesized as previously described.^33^ Amine‐functionalized particles (TiO_2_‐NH_3_^+^) were prepared by adding a solution of *N*‐(3,4‐dihydroxyphenyl)acetimide (25 mg in 2 mL ethanol) drop‐wise into a cyclohexane suspension of oleic acid‐modified TiO_2_ nanocrystals (2 mL, 0.7 wt %). Triethylamine (200 mg) was added and the mixture was stirred at the room temperature for 2 h to complete the ligand exchange. The nanocrystals were collected by centrifugation and then dispersed in ethanol (4 mL) with aqueous KOH (5 M, 50 μL) and stirred for 1 h to hydrolyze the amide bonds to amine functional groups. Ethanol (3 mL) was added and the particles were collected by centrifugation. Finally, the particles were washed with a mixture of acetone (5 mL) and water (1 mL) before dispersing in water (3 mL). Dye‐sensitized **RuP**‐TiO_2_‐NH_3_^+^ particles were prepared by adding an aqueous solution of **RuP** (1 mM) to the solution of TiO_2_‐NH_3_^+^ as previously described for **RuP**‐TiO_2_‐COO^−^.^33^

### Synthesis of CdS Nanoparticles

Hydrophobic, oleic acid‐capped CdS QDs (CdS‐OA) were prepared by a standard hot injection method^44^ and positive (CdS‐N(CH_3_)_2_H^+^) or negatively (CdS‐COO^−^) charged ligands were introduced by previously reported ligand exchange procedures.^45^, ^46^

**CdS‐OA** Briefly, CdO (0.64 g) and OA (29 mL) were suspended in ODE (89 mL) and heated under an Ar atmosphere to 280 °C. A solution of sulfur (0.08 g in 24 mL ODE) was added rapidly and the reaction was continued for 2 min before quenching in a water bath. The particles were isolated using 1 : 1 hexane:methanol (100 mL) and excess acetone. After centrifugation, the particles were washed a further two times using hexane and acetone as solvent and non‐solvent, respectively, before suspending in hexane (20 mL).

**CdS‐COO^−^** CdS‐OA solution (2 mL) was added to a solution of MPA (0.5 mL in 10 mL 1:1 methanol:chloroform) at pH 11. The solution was stirred for at least 16 h before isolating CdS‐COO^−^ by centrifugation (5000 g, 5 min) and washing with methanol and acetone as solvent and non‐solvent, respectively. The final precipitate was suspended in deionised water (1 mL).

**CdS‐N(CH_3_)_2_H^+^** CdS‐OA solution (1 mL) was added to a Schlenk flask and the solvent removed. The particles were re‐suspended in CHCl_3_ (0.5 mL) under Ar, and a solution of DMAET (1 mL, 1 M in methanol) was added. The mixture was protected from light and stirred vigorously for 16 h. The particles were precipitated with excess acetone and centrifuged (5000 g, 5 min). The particles were washed a further two times before suspending in deionised water (0.5 mL).

### Characterization of RuP‐TiO_2_ and CdS Nanoparticles

Transmission electron microscopy (TEM; HitachiH7650, Hitachi) was used to record high resolution images at 200 keV. X‐ray diffraction (XRD) patterns were obtained with either a Rigaku D/Max‐2500 or an X′Pert PRO by PANalytical BV diffractometer using Cu Kα radiation (λ=1.5418 Å) at a scanning rate of 4.00° min^−1^. Zeta potential measurements were carried out using a Malvern Instruments nanocomposite size analyzer (NanoZS, Worcestershire, UK). Fourier‐transform infrared (FT‐IR) spectra were obtained using a Thermo Scientific Nicolet iS50 FTIR spectrometer in ATR mode. UV‐vis spectroscopy was carried out using a Varian Cary 50 UV‐Vis spectrophotometer. Thermal gravimetry (TG) analysis (TG8120, Rigaku) was performed to estimate the quantity of the modifiers or molecules that attached on the surfaces of the TiO_2_‐NH_3_^+^ nanocrystals. 2.03 mg of the TiO_2_‐NH_3_^+^ nanocrystals were loaded for TG analysis. Temperature was maintained at 150 °C for 2 h for dehydration and then raised to 800 °C at the rate of 20 °C min^−1^.

### Electrode Preparation and Protein‐Film Voltammetry

Template stripped gold (TSG) was prepared as described previously.^47^ Briefly, 150 nm gold (99.95 %; Goodfellow) was evaporated on silicon wafers (IDB Technology Ltd, UK) using an Edwards Auto 306. After evaporation, 1.2 cm^2^ glass slides were glued to the gold layer with Epo‐Tek 377, for 2 hours at 120 °C. Self‐assembled monolayers (SAMs) were made by incubating freshly detached glass slides, exposing the TSG surface, with either 0.8 mM 8‐mercaptooctanol/0.2 mM 8‐amino‐1‐octanethiol or 0.8 mM 6‐mercaptohexanol/0.2 mM 6‐amino‐1‐hexanethiol in water for a minimum of 2 days at 4 °C. After incubation, excess thiol was gently washed away with water and the electrode was dried under a nitrogen flow.

For protein film electrochemistry, a home‐build electrochemical cell was used with a standard three‐electrode setup. The SAM‐modified TSG was embedded in a polytetrafluoroethylene (PTFE) holder with a rubber O‐ring seal, placed in a glass electrochemical cell container as the working electrode. The counter electrode was a platinum wire and the reference electrode was a saturated mercury/mercury sulfate electrode (Hg/HgSO_4_; Radiometer analytical, France). All potentials are quotes versus standard hydrogen electrode (SHE) using 0.649 mV vs SHE for the Hg/HgSO_4_ reference electrode. After measuring ‘blank’ cyclic voltammograms (CVs) in 2 mL electrolyte buffer (20 mM MOPS, 30 mM Na_2_SO_4_ at pH 7.4), the buffer was removed and 50 μL of MtrC (0.87 μM) or OmcA (1.2 μM) protein in buffer was directly added to the working electrode surface and incubated for 1 min at 20 °C. The electrode was rinsed more than three times with 2 mL buffer taking care to retain the electrode under fluid at all times. CVs were measured in fresh buffer (2 mL) at 20 °C using an Autolab electrochemical analyzer (Eco‐chemie, Utrecht, Netherlands) equipped with a PGSTAT 128N potentiostat, SCANGEN and ADC10M modules, and FRA2 frequency analyzer (Ecochemie). CV experiments were routinely carried out by holding the potential at 0.19 V for 5 s before cycling at a scan rate of 1 V s^−1^ in the potential window from 400 mV to −500 mV (vs SHE). Analysis was performed with the freely available software Q‐Soas.^48^ To minimize electrical noise all experiments were conducted in a steel mesh Faraday cage, and argon purging was performed to avoid oxygen reduction.

### Quartz Crystal Microbalance with Dissipation (QCM‐D)

QCM−D measurements were performed using a Q‐Sense E4 (Q‐Sense AB). Gold‐coated QCM−D crystals were cleaned with 2 wt % SDS detergent for 10 min using bath sonication, rinsed with water and dried under a nitrogen flow. QCM−D crystals were subsequently treated for 20 min with UV/ozone (UVOCS Inc T10x10/OES/E, UK). Gold oxide, formed on the surface by the ozone treatment was reduced by incubating the crystals for 30 min in freshly distilled propanol at 40–60 °C. Freshly cleaned QCM−D crystals were modified with different SAM solution as above for 2 days at 4 °C and then rinsed with water. QCM−D experiments were conducted at 21 °C, with the flow rate held at 70 μL min^−1^. All protein‐binding experiments were performed in buffer (20 mM MOPS, 30 mM Na_2_SO_4_ at pH 7.4). Incubations with OmcA or MtrC (1 μM) and **RuP**‐TiO_2_ or CdS (0.2 mg mL^−1^) were performed as indicated in the result section, where changes in the dissipation (ΔD) and frequency (Δ*f*) of the third overtone are presented. Fifth, seventh, ninth, eleventh, and thirteenth overtones were also recorded. The binding coverage of the proteins and nanoparticles was estimated by using the Sauerbrey equation (i. e., 17.7 ng cm^−1^ Hz^−1^ for the equipment and crystals used).

### Construction and Characterization of OmcA/RuP‐TiO_2_ and OmcA/CdS

To adsorb **RuP**‐TiO_2_ or CdS on electrodes modified with OmcA or MtrC in the electrochemical setup (see above), the electrolyte solution was almost removed without drying the protein film and 50 μL **RuP**‐TiO_2_ nanocrystals or CdS nanoparticles (typically 0.2–0.5 mg mL^−1^ in 25 mM EDTA or 25 mM TEOA) was added. The same procedure was also performed on SAM modified electrode without OmcA/MtrC. After incubation for 5–10 min, the electrochemical cell was rinsed several times to remove any unbound **RuP**‐TiO_2_ or CdS.

A cold light source (Krüss KL5125, Germany) with a 150 W, 4.5 cm (15 V) halogen lamp (OSRAM) and fiber optic was placed 5–10 cm above the gold electrode with the light passing through ∼2 cm of buffer before reaching the electrode surface. The light intensity on the electrode (area=0.25 cm^2^) was measured separately and found to be approximately 50 mW (i. e., 200 mW cm^−2^). Photoelectrochemical measurements were performed by measuring photogenerated currents between the modified working electrodes and a Pt counter electrode using an Autolab electrochemical analyzer, as described above. To confirm that light source did not directly excite the TiO_2_ with ultraviolet emissions, control experiments were performed with a UV filter (cut‐off 375 nm), which showed no difference in photocurrents. Linear sweep voltammetry (LSV) was used to determine the photoelectrochemical properties of all samples under controlled illumination. The scan rate was 5 mV s^−1^ between −450 mV and +450 mV versus SHE. For chronoamperometry measurements, the potential was set at +400 mV versus SHE and the electrode was typically illuminated for 10 s (40 s off period). Chronoamperograms and voltammograms were baseline corrected (during the dark phases) with Q‐Soas.^48^ All experiments were performed at 19±2 °C.

## Conflict of interest

The authors declare no conflict of interest.

## Supporting information

As a service to our authors and readers, this journal provides supporting information supplied by the authors. Such materials are peer reviewed and may be re‐organized for online delivery, but are not copy‐edited or typeset. Technical support issues arising from supporting information (other than missing files) should be addressed to the authors.

Supplementary
