# Supplementary material for: Exploring Step‐by‐Step Assembly of Nanoparticle:Cytochrome Biohybrid Photoanodes
Source: ChemElectroChem. 2017 May 15;4(8):1959–68. doi: 10.1002/celc.201700030 (PMC5573906; doi:10.1002/celc.201700030)
Supplement: Supplementary file 1 — Supplementary [file CELC-4-1959-s001.pdf]

Supporting Information

© Copyright Wiley-VCH Verlag GmbH & Co. KGaA, 69451 Weinheim, 2017

Exploring Step-by-Step Assembly of Nanoparticle:Cytochrome Biohybrid Photoanodes

Ee Taek Hwang, Katherine L. Orchard, Daisuke Hojo, Joseph Beton, Colin W. J. Lockwood, Tadafumi Adschiri, Julea N. Butt,* Erwin Reisner,* and Lars J. C. Jeuken*© 2017 The Authors. Published by Wiley-VCH Verlag GmbH & Co. KGaA. This is an open access article under the terms of the Creative Commons Attribution License, which permits use, distribution and reproduction in any medium, provided the original work is properly cited.

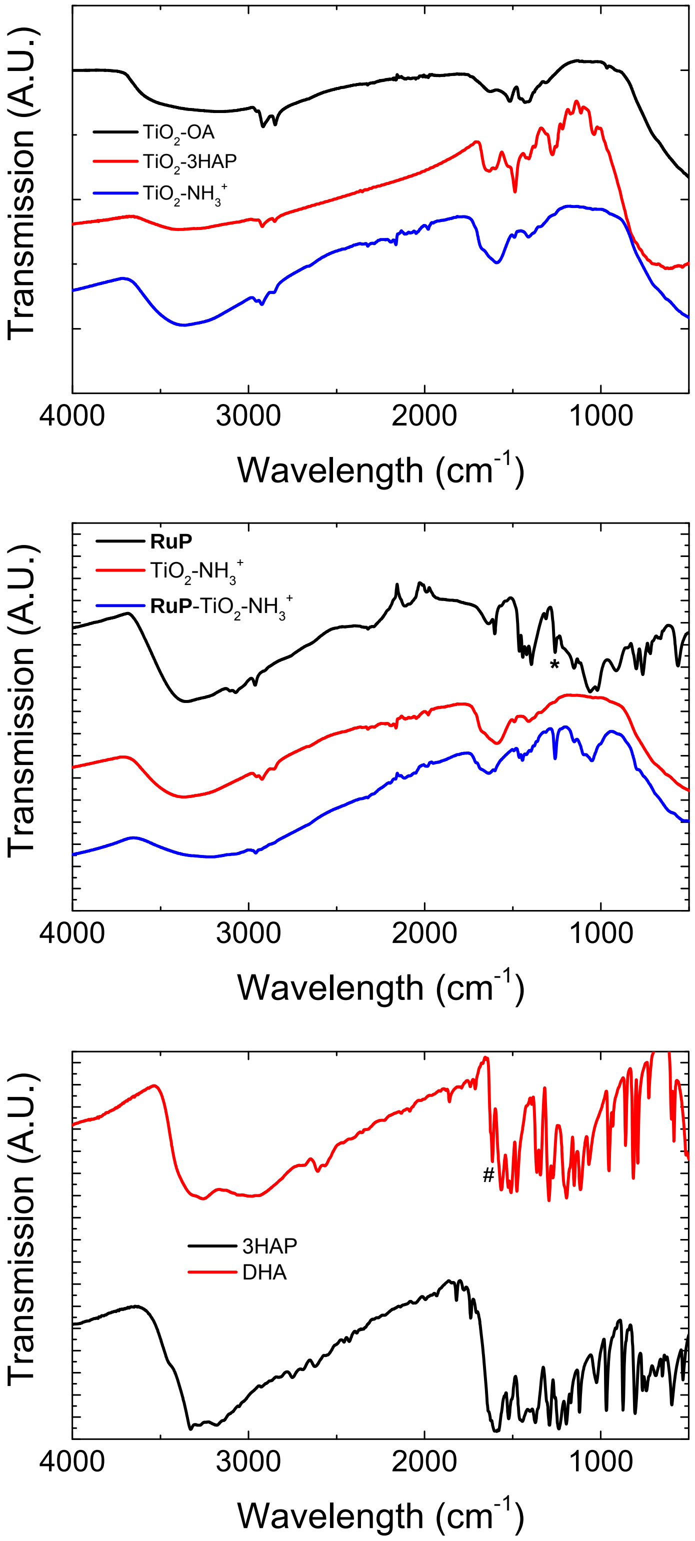

Figure S1. (Top) Fourier-transform infrared (FT-IR) spectra of TiO_2-OA (oleic acid), TiO_2-3HAP and TiO_2-NH_3^+ (= TiO_2-DHA), as indicated. (Middle) FT-IR spectra of **RuP**, TiO_2-NH_3^+ (= TiO_2-DHA) and **RuP**-TiO_2-NH_3^+, as indicated. The peak marked with “*” correspond to the phosphonate groups of **RuP**. (Bottom) Reference FT-IR spectra of 3HAP and DHA. The peak marked with “#” correspond to the N-H vibration.

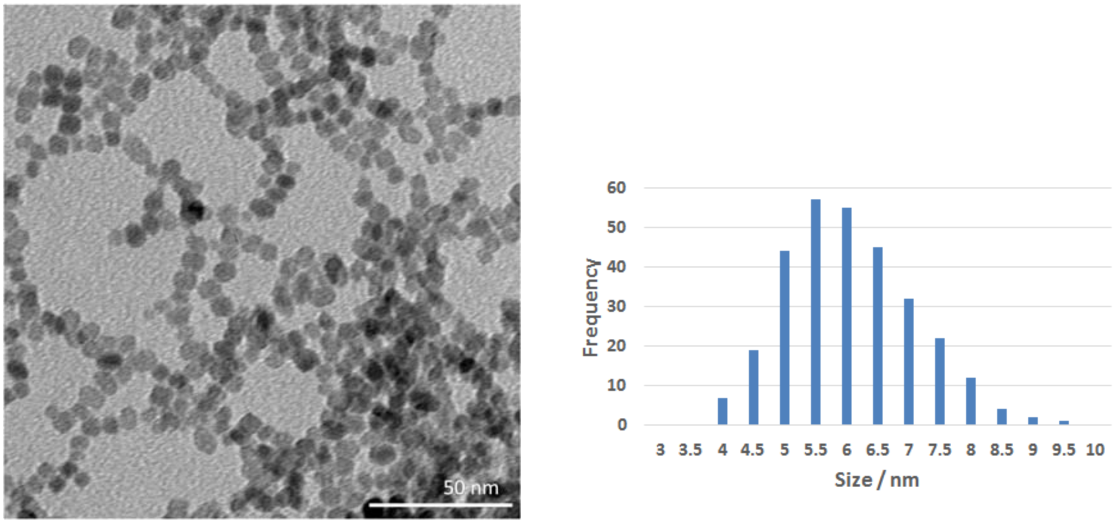

Figure S2. TEM image (Left) and size distribution histogram (Right) of the TiO_2-NH_3^+ nanocrystals. The histogram was prepared from the measurements of 300 nanocrystals randomly taken from the TEM images. The size of the TiO_2-NH_3^+ nanocrystals was evaluated to be 5.8 ± 1.0 nm. This relatively large size distribution was due to the shape of the nanocrystals, which deviate from a perfect spherical shape; the apparent size distribution is increased because two-dimensional projection image is taken from TEM.

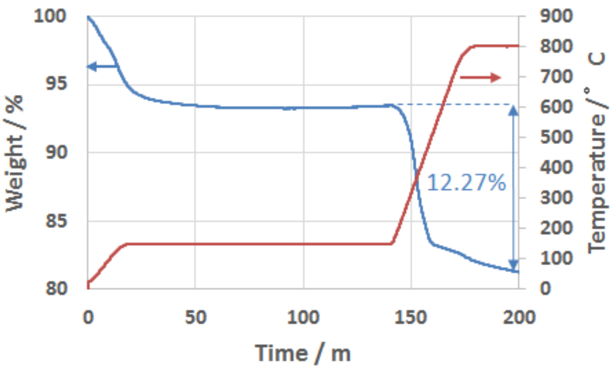

Figure S3. Thermal gravimetry (TG) analysis of freeze-dried TiO_2-NH_3^+ nanocrystals. Weight % and the temperature are plotted versus heating time (in min).

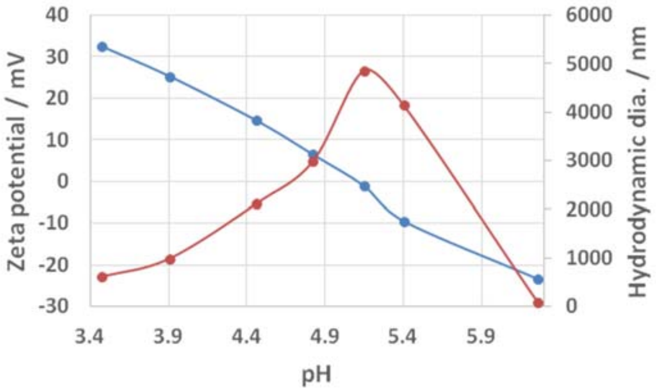

Figure S4: Zeta potential (blue circles, left axis) and hydrodynamic diameter (red circles, right axis) of TiO_2-NH_3^+ nanocrystals in aqueous solution. The isoelectric point is determined at pH 5.1, coinciding with aggregation of the nanoparticles as suggested by the increase in hydrodynamic radius.

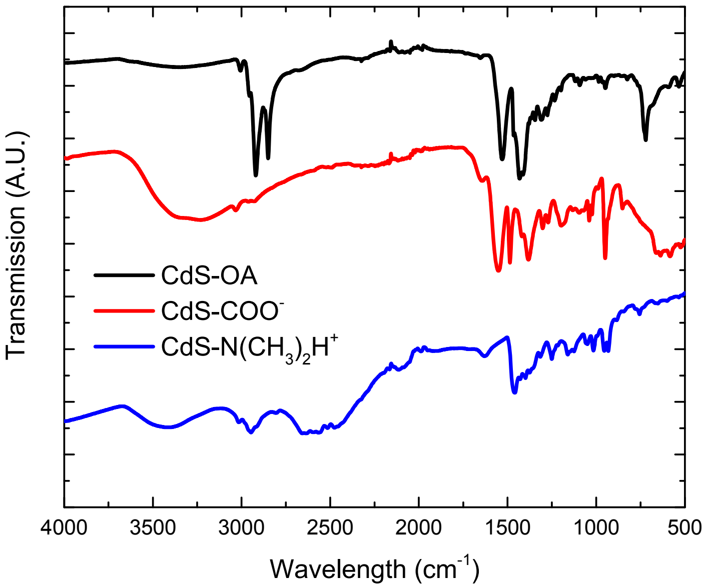

Figure S5. FT-IR spectra of CdS-OA, CdS-COO^-, and CdS-$N(CH_3)_2H^+$.

(A)

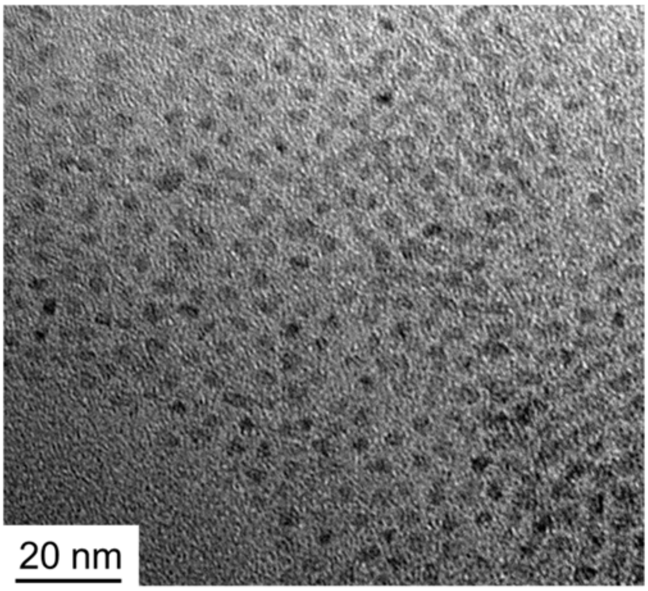

(B)

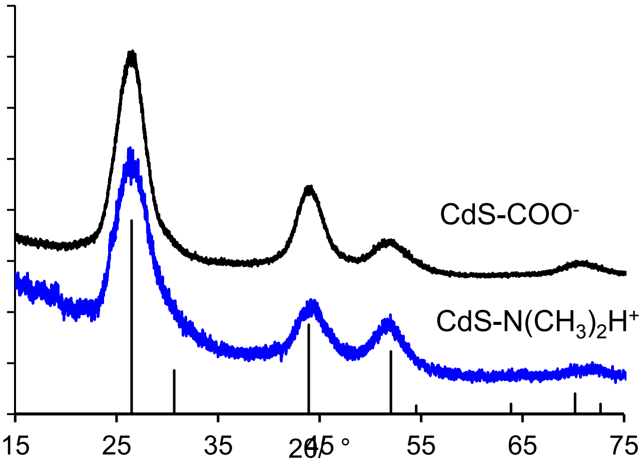

Figure S6. TEM images of (A) CdS-OA as synthesised, and (B) X-ray diffraction (XRD) pattern of CdS-COO^- (black line) and CdS-$N(CH_3)_2H^+$ (blue line), compared with the reference CdS spectrum (black, PDF no. 01-080-0019 10-454).

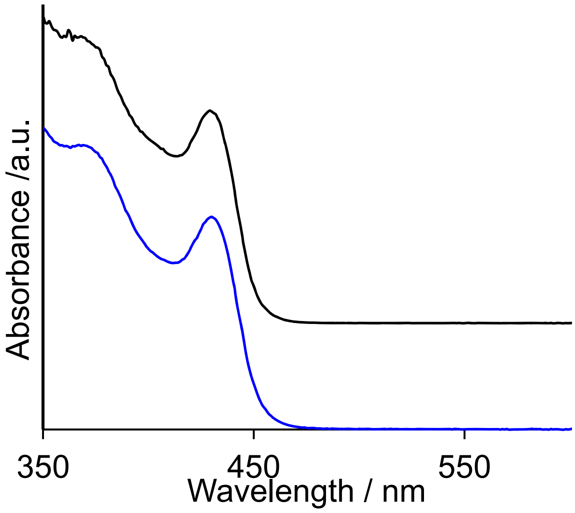

Figure S7. Solution phase UV-Vis absorption spectra of as-synthesised CdS quantum dots: CdS-COO^- (black line) and CdS-$N(CH_3)_2H^+$ (blue line) in water.

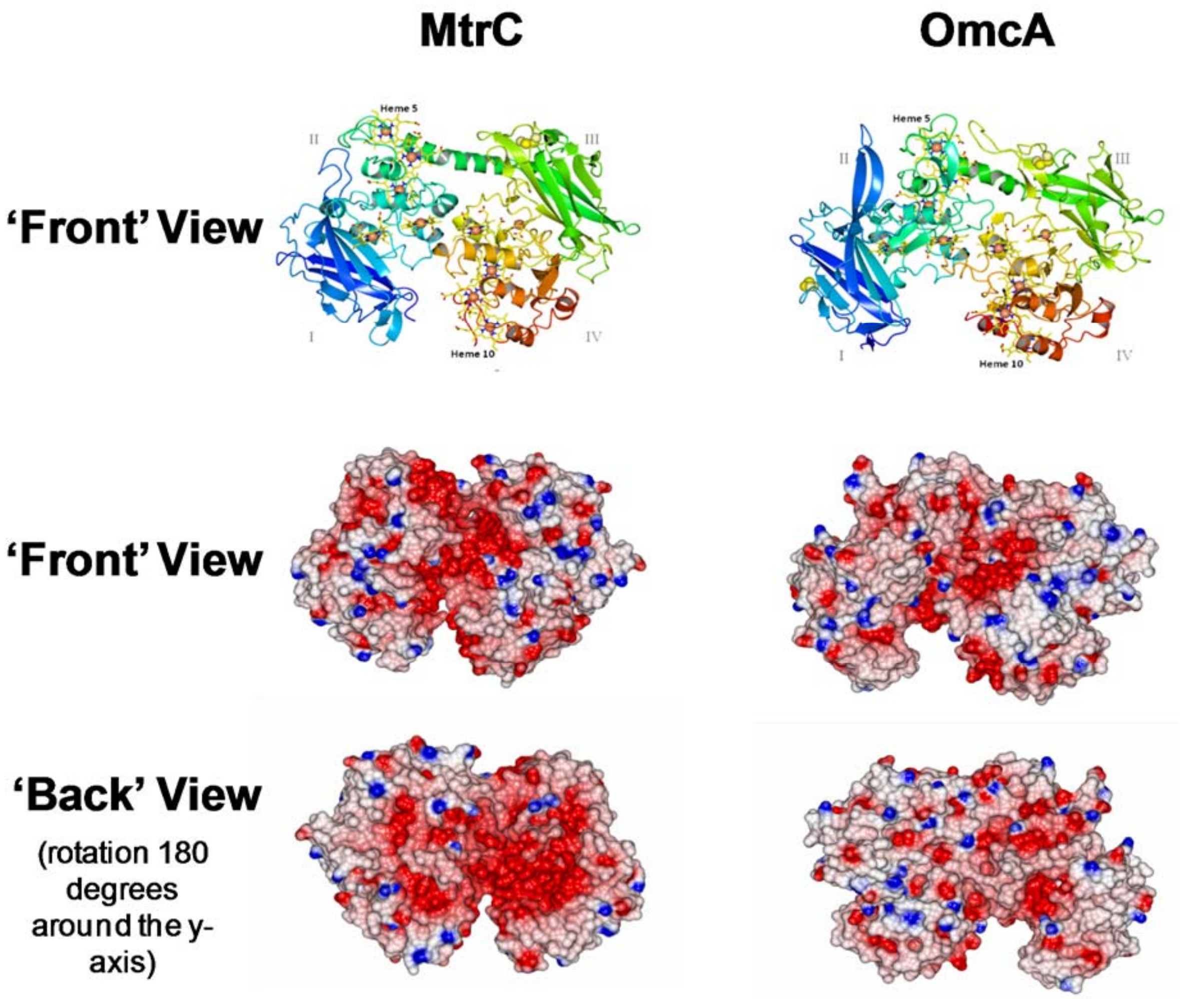

Figure S8. Electrostatic surface maps for MtrC[1] and OmcA[2] calculated using CCP4mg[3], with potentials scaled from −0.5 V (red, negatively charged) to +0.5 V (blue, positively charged).

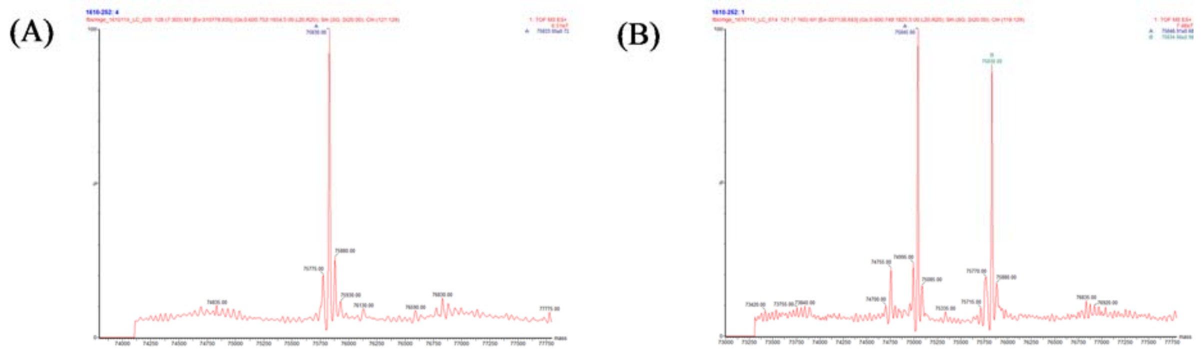

(C) C-terminus of the Engineered MtrC:

ADHTKVKM/*/DDDDKLAACCPGCCKKGELKLEGKPIPNPLLGLDSTRTGHHHHHH

Figure S9. (A) LC-MS results of purified MtrC with the mass of the main fraction at 75,830 Da. MtrC preparations with this mass displayed the electroactive behavior reported in the main manuscript with an electroactive coverage of 0.17±0.02 pmol/cm^2 (B) LC-MS result of an MtrC preparation that resulted in a very low electroactive coverage (<0.02 pmol/cm^2). Two fractions are visible and the main fraction has a (lower) mass of 75,045 Da. (C) The C-terminus of the engineered MtrC sequence with the non-native, engineered sequence in red. The calculated MW of the full MtrC protein (with engineered sequence) is 79,925 Da, without the engineered sequence is 75,047 Da and cleaved after the lysine in the enterokinase protease sequence (underlined) the calculated MW is 75,636 Da.

References

1. Edwards, M. J.; White, G. F.; Norman, M.; Tome-Fernandez, A.; Ainsworth, E.; Shi, L.; Fredrickson, J. K.; Zachara, J. M.; Butt, J. N.; Richardson, D. J.; Clarke, T. A., Redox Linked Flavin Sites in Extracellular Decaheme Proteins Involved in Microbe-Mineral Electron Transfer. *Sci. Rep.* **2015,** *5*, Artn. 11677.
2. Edwards, M. J.; Baiden, N. A.; Johs, A.; Tomanicek, S. J.; Liang, L. Y.; Shi, L.; Fredrickson, J. K.; Zachara, J. M.; Gates, A. J.; Butt, J. N.; Richardson, D. J.; Clarke, T. A., The X-ray crystal structure of Shewanella oneidensis OmcA reveals new insight at the microbe-mineral interface. *Febs Lett.* **2014,** *588* (10), 1886-1890.
3. McNicholas, S.; Potterton, E.; Wilson, K. S.; Noble, M. E. M., Presenting your structures: the CCP4mg molecular-graphics software. *Acta Crystallogr. D* **2011,** *67*, 386-394.
